# Application of the Theory of Planned Behavior to couples' fertility decision-making in Inner Mongolia, China

**DOI:** 10.1371/journal.pone.0221526

**Published:** 2019-08-23

**Authors:** Xinhua Li, Yancun Fan, Sawitri Assanangkornchai, Edward B. McNeil

**Affiliations:** 1 Faculty of Health Management, Inner Mongolia Medical University, Hohhot, Inner Mongolia, China; 2 Epidemiology Unit, Faculty of Medicine, Prince of Songkla University, Hat Yai, Songkhla, Thailand; Middlesex University, UNITED KINGDOM

## Abstract

China relaxed its family planning policy and adopted a universal two-child policy on January 1, 2016 to actively address the country’s aging trend. However, the policy has failed to have any significant effect on the fertility rate of many provinces. In light of the country having the highest sex ratio at birth in the world and the huge burden of the aging population, improving the fertility rate is an urgent priority in China. This facility-based cross-sectional survey aimed to study determinants of fertility decision-making among couples based on the Theory of Planned Behavior. The study was conducted in Inner Mongolia Autonomous Region of China. A structured self-administered questionnaire was completed by 1,399 couples, consisting of wives aged 20–49 years and their husbands. Based on the structural equation modeling method of analysis, determinants of fertility decision-making were perceived behavior control (perceived importance of having a stable income and cost of raising a child), subjective norms (perceived social pressure about “sex preference of the newborn by themselves and their partner”) and attitudes (only healthy parents can have a child). Other significant factors influencing fertility decision were ethnicity and education level, with ethnic minority couples having less perception of social norm towards fertility and those with higher education having higher perceived control toward having a (further) child. The study reveals the importance of the China’s infrastructure and public facilities to support child-rearing to increase the fertility rate among couples of child-bearing age, which in turn will reduce the burden associated with an aging society.

## Introduction

Since the 1960s, global fertility rates have halved, grabbing the attention of researchers. Low fertility rates are now common in both developed and developing countries [1–4]. The situation has raised the question of whether fertility behavior adequately reflects people’s preference for the number of children they would like to have; the discrepancy between ideal and actual number of children known as the fertility gap [5].

On January 1, 2016 China relaxed its family planning policy and adopted a universal two-child policy to actively address the country’s aging trend. However, the new policy has failed to have any significant effect on the fertility rate of many provinces [6, 7], and in fact the birth rate decreased from 13.0 births/1,000 women in 2016 to 12.3 births/1,000 women in 2017 [6]. In light of the country having the highest sex ratio at birth in the world and the huge burden of the aging population [8], improving the fertility rate is an urgent priority in China. Motivated by the above scenario, an understanding of the determinants of fertility intention will help policy-makers tackle these challenges.

Although Inner Mongolia is an Autonomous Region of China which has relaxed the one-child policy for Mongolian people, the birth rate of Inner Mongolia has been very low for many years. In 2015, the birth rate in Inner Mongolia was 7.72‰ which ranks it 26^th^ among the 34 provinces of China; at the same time the birth rate was 12.07‰ in the whole country [9]. This study aims to determine the psychosocial determinants on a couple’s fertility intention that influences the ‘fertility gap’ and to understand how couples make their fertility intention within the opportunities and constrains provided by the societal structures in which they are embedded. The discrepancy between ideal number and planned number of children is largely unexplored and calls for further research on fertility decision-making among couples.

Wives’ age at marriage is one of the most important determinants of child-birth in a family. Under the Chinese law regarding marriage and birth, the postponement of marriage means the postponement of childbirth. In addition, the out-of-wedlock birth is in conflict with traditional Chinese culture, so any level of fertility in a population can always be traced by the percentage of women at first age of marriage [10, 11].

Minorities in China have had low fertility rates since the 1990s [12], even being supported by the relative liberal one child policy in China. Inner Mongolia has the lowest fertility rate in all Autonomous Regions in China [13], in both urban and rural areas. Compared with Han, the Mongolian ethnic group have fewer sons and a multi-child preference [4].

Most research studies combined demographic characteristics, such as education, occupation, and income, as socioeconomic factors linked with fertility [14–16]. Previous research showed a significant negative correlation between fertility and wives’ socioeconomic status [17], but the negative relationship disappears or becomes even positive after accounting for the endogeneity of schooling [18, 19]. A study from Norway showed that husbands who had a higher education delayed their fatherhood, yet fewer remained childless, and the rates of second and third births increased with their educational attainment [20]. Among all published studies, the impact of husbands’ socioeconomic status on fertility decision has largely been overlooked. Similar situations were reported in studies from China [21–23].

The Theory of Planned Behavior (TPB) is an extensively validated psychosocial theory useful for understanding couples’ fertility decision [24]. It is a general psychological theory concerning the link between attitudes and behaviors and has also been applied to explain fertility decision-making [25–27]. Based on the TPB, three determinants influence behavior intention which is a proxy measure of the behavior (in our case, a decision to have a child): 1. attitudes toward the behavior which refer to the individual’s positive and negative feeling of the behavior and outcome of performing the behavior; 2. subjective norms, which relates to the individual’s perception of the social environment surrounding the behavior; and 3. perceived control over the performance of the behavior [24].

**Fig 1:**
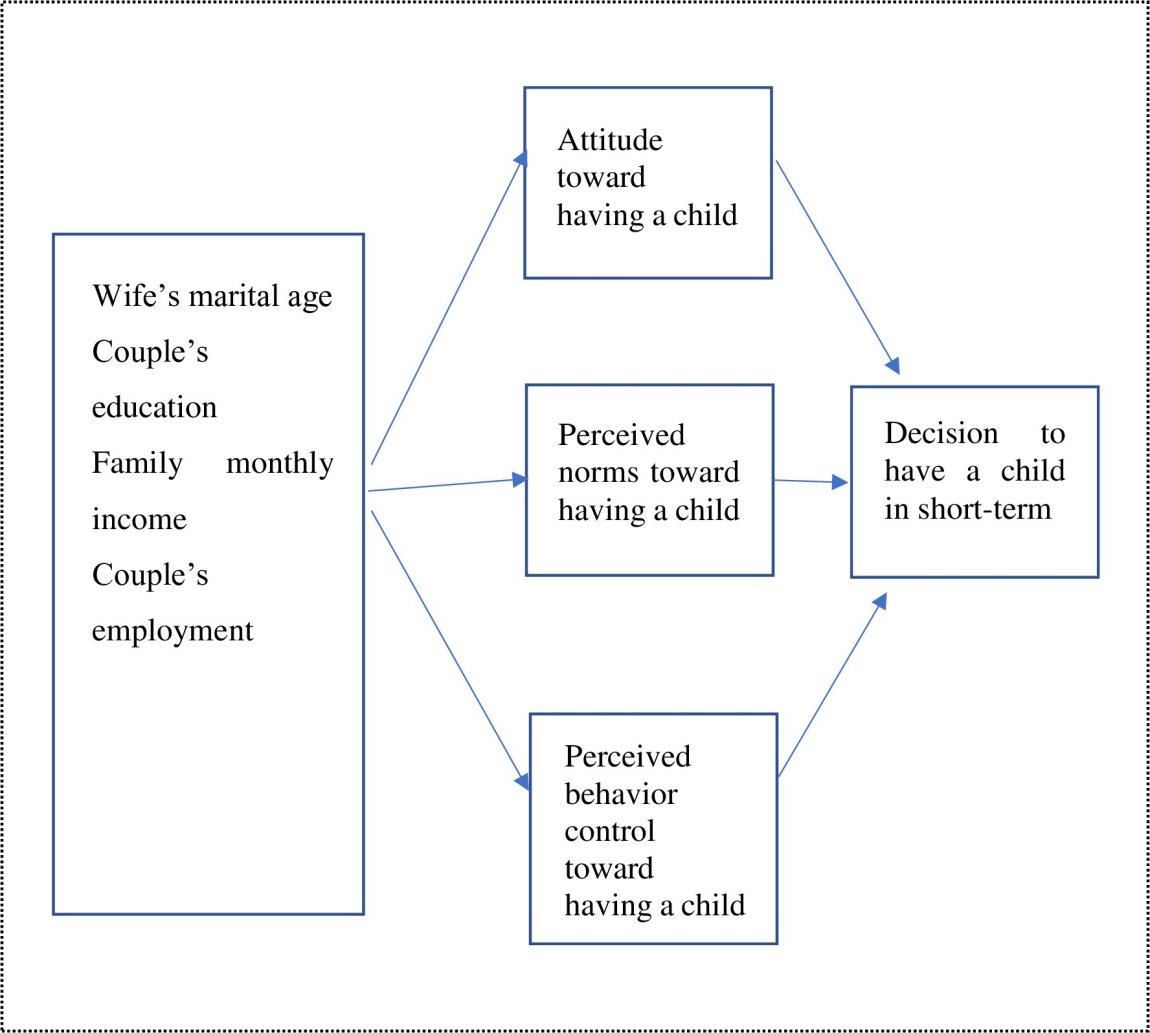
Conceptual model based on the Theory of Planned Behavior.

The objective of this research was to investigate determinants of fertility decision-making among couples in Inner Mongolia. We developed our initial conceptual model based on the Theory of Planned Behavior [27] (Fig 1).

## Material and methods

### Participants, recruitment setting and sampling procedure

A facility-based cross-sectional survey was conducted in Xin Cheng, an urban county in Hohhot district and Zhuo Zi, a rural county in Ulanqab district of Inner-Mongolia province of China between March and July 2018.

Considering that the estimated proportion of eligible couples applying for permission to have a second child was 0.13 [28], to estimate the proportion with a precision of 5%, and allowing for 20% nonresponse rate, and a design effect of 2 for cluster sampling, the sample size required for this study was 1,305 (174/0.8×2×3). In each county, data were collected from three different settings: marriage registration offices, antenatal care (ANC) clinics, and kindergartens.

All Chinese couples in which the wife was aged 20–49 years and both were an Inner Mongolian citizen who visited one of the above settings were eligible for the study. At marriage registration offices, couples who had just married and had no children were recruited. At the ANC clinics, pregnant women were recruited, while at kindergartens, couples who had already had one or two children were recruited. The minimum age of 20 years for women was used because this is the age at which Chinese women can legally be married in China.

### Measures

#### Social and demographic variables

Data on demographic characteristics and psychosocial determinants on fertility decision were derived from a structured self-administered questionnaire. Demographic variables included wife’s age at marriage, couples’ education levels (low: both senior high school or below, middle: at least one college degree, high: at least one above college degree), ethnicity (at least one ethnic minority, or both Han), family monthly income, and occupation (both self-employed, at least one self-employed, or both employed).

#### Predictors of fertility decision

The dependent variable was fertility decision, measured by a question: “what is your family plan in the next 2 to 3 years?”. The possible responses were: (1) I will have a (another) child in 2–3 years, (2) I already have two children, (3) no plan for any child, (4) one child is enough for me. Factors predicting fertility intention was measured using a modified TPB questionnaire [27]. Questions were developed according to the main constructs of the TPB [29] in the context of fertility decision-making using the guideline provided in the TPB manual [30]. This questionnaire was developed after performing a literature review and conducting in-depth interviews with a panel of experts who assessed the questionnaire’s validity. The panel of experts included one health policy researcher, one psychologist and two epidemiologists. A pilot study was conducted before the survey to ensure feasibility of the study and to test the questionnaire, which was modified after the pilot study and finalized by the team of experts.

The questionnaire included three dimensions with 13 individual items:

1. Perceived behavior control to have a (another) child (PC). This dimension was assessed with six statements: PC1: the couple is ready to sacrifice the time and freedom for the baby; PC2: a suitable babysitter is available when the couple works outside; PC3: a lot of money will be spent to raise a (another) baby; PC4: the couple has sufficient materials for child rearing; PC5: the couple’s family members can help them take care of the baby; PC6: the couple has a stable income.
2. Subjective norms regarding having a (another) child (SN). This dimension was assessed with three statements related to the couple’s perceived social pressure on their fertility decision. The statements are as follows, SN1: the family should have a boy (or girl); SN2: his/her partner prefers to have a boy (or girl); SN3: the relatives and friends around the couple have already had two children.
3. Attitudes towards having a (another) child (AT). This dimension was assessed with four statements related to the couple’s attitudes towards having a child. The statements are as follows, AT1: the couple enjoys having a big family; AT2: the couple enjoys the fun of raising a baby; AT3: Only healthy parents can have a child; AT4: having a child can maintain a good relationship between the couple. The responses of each statement was based on the forced choice method (no neutral choice) [31], which is rated on a four-point scale (1 = not important at all, 2 = of little importance, 3 = very important, and 4 = absolutely essential). The total score of the whole fertility decision model was constructed by adding the scores of all items together, yielding a possible range of scores between 13 and 52.

#### Ethical approval and consent to participate

Before collecting the data, written informed consent was obtained from all participants. The study protocol was approved by the Research Ethics Committee of the Faculty of Medicine, Prince of Songkla University (reference number: 60-429-18-1). No additional ethical approval was required from the three study sites.

#### Data management and statistical analysis

Data were entered and validated using Epidata, version 3.1. R version 3.4.4 was used for data analysis. Amos21.0 was used to fit the structural equation models. No question was shown to contain any missing data. Three age groups were created: “< 24 years”, “24–35 years”, and “36–49 years”. The cut-point of 24 years was considered the age for college matriculation and 35 years the maximum safe age for parturient women.

Means and standards deviation were used to describe the manifest variables of the TPB model of fertility decision. The total score of the model was calculated from the sum of the scores of responses to all 13 manifest variables. Comparison of the total scores of the fertility decision model across socio-demographic variables were performed using the Kruskal-Wallis test. Structural equation models (SEM) with maximum likelihood estimation [32] was used to explore the relationship between variables. All 13 manifest variables and social-demographic variables with a p-value less than 0.2 from the Kruskal-Wallis test were included in the initial structural equation prototype model. Standardized regression weights (β) were obtained from the SEM, indicating the total effect of each manifest variable on the outcome variable (fertility decision). As recommended by Anderson and Gerbing [33], goodness of Chi-square statistic, root mean square error of approximation (RMSEA), adjusted goodness fit index (AGFI), and comparative fit index (CFI) were used to choose the best fitting model. Cronbach’s alpha was used to test the reliability of scales, a value greater than 0.70 was regarded as satisfactory [34].

## Results

### Demographic characteristics

A total of 1,513 couples were asked to join the study, of which 1,399 (92.5%) agreed to participate and completed the whole questionnaire and whose data were included in the analysis. The mean age at marriage of the wives was 26.3 years (standard deviation (SD) = 3.1). The majority of the families (74.3%) belonged to the Han ethnicity and most had a middle level education. About one-third of the couples (34.2%) were both self-employed. Over 80% of the couples had monthly income less than 10,000 Chinese yuan (equal to 1,440 US dollars) (Table 1).

**Table 1:** Demographic characteristics of the study sample.

Characteristic	Frequency	%
Ethnic group		
At least one minority	359	25.7
Both Han	1040	74.3
Education level		
Both junior college and below	566	40.5
At least one college	646	46.2
At least one above college	187	13.4
Employment status		
Both self-employed	479	34.2
Only one self-employed	346	24.7
Both employed	574	41.0
Monthly family income (RMB)		
≤ 3000	156	11.2
3001–5999	445	31.8
6000–9999	482	34.5
10001–19999	247	17.7
≥ 20000	69	4.9
Wife's age at marriage		
≤ 23	234	16.7
24–35	1151	82.3
36–47	14	1

RMB: Chinese renminbi.

### Descriptive analysis of construct variables of fertility decision model

Summary statistics of all variables of the three dimensions in the fertility decision model are shown in Table 2. The mean overall score was highest for the Perceived behavior control domain (2.52), followed by Attitudes (2.34), and Subjective norm (1.89). The means of all individual items were in the middle range (1.86–2.68), indicating that most respondents placed the value of each factor as “of little importance” to “very important”. Cronbach’s alphas of all domains exceeded the minimum acceptable level of 0.70, indicating high internal consistency of the items measuring the same construct.

**Table 2:** Summary of behavioral constructs and their subdomains.

Measure	Range	Mean	S.D.	Cronbach’s alpha
Perceived behavior control (PC)	1–4	2.52	1.17	0.859
PC1	1–4	2.36	1.01	
PC2	1–4	2.46	1.11	
PC3	1–4	2.68	1.03	
PC4	1–4	2.62	1.07	
PC5	1–4	2.57	1.13	
PC6	1–4	2.47	1.11	
Subjective norms (SN)	1–4	1.89	0.82	0.707
SN1	1–4	1.86	0.93	
SN2	1–4	1.88	0.93	
SN3	1–4	1.93	0.86	
Attitudes (AT)	1–4	2.34	1.34	0.829
AT1	1–4	2.27	1.08	
AT2	1–4	2.41	1.10	
AT3	1–4	2.35	1.23	
AT4	1–4	2.33	1.22	

Legend of the variables

SD: standard deviation.

PC1: The couple is ready to sacrifice the time and freedom for the baby

PC2: A suitable babysitter is available when the couple works outside

PC3: A lot of money will be spent to raise a (another) child

PC4: The couple has sufficient materials for child rearing

PC5: The couple’s family members can help them take care of the baby

PC6: The couple has a stable income

SN1: The family should have a boy (or girl)

SN2: His/her partner prefers to have a boy (or girl)

SN3: The relatives and friends around the couple have already had two children

AT1: The couple enjoys having a big family

AT2: The couple enjoys the fun of raising a child

AT3: Only healthy parents can have a child

AT4: Having a child can maintain a good relationship between the couple

### Comparison of the total scores of the fertility decision model by socio-demographic characteristics

Table 3 shows the total scores of all manifest variables of the fertility decision model by social-demographic variables. Higher scores indicate a higher perception of the importance of perceived behavior control, social norm and positive attitudes on the decision to have a (another) child. The results showed that the scores of fertility decision were not different between couples belonging to minority and Han ethnic groups. However, significantly higher score were seen among more educated couples, couples with a higher monthly family income and those who were both employed, compared to their counterparts. Wives who married between the ages of 24–35 years had higher mean scores compared with those married at an earlier or later age. Based on these univariate results, education, employment status, monthly income and wife’s age at marriage were included in the initial SEM of the fertility decision model. Although ethnicity was not significantly associated with the fertility decision in this univariate analysis, it was deemed to be an important factor for the fertility decision (the fertility policy has been regulated differently between Han majority and other ethnic minority populations), it was thus kept in the initial model.

**Table 3:** Comparison of the total scores of the fertility decision model by socio-demographic variables.

Determinant	Score of fertility decision model Median (IQR)	p-value
Ethnic group		0.46
At least one minority	31 (24,38)	
Both Han	31 (23,37)	
Education level		<0.001
Both junior college and below	27 (20,35)	
At least one college	32 (24,38)	
At least one above college	36 (29,40)	
Employment		<0.001
Both self-employed	27 (20,34)	
Only one self-employed	32 (22,38)	
Both employed	34 (26,39)	
Monthly income (RMB)		0.001
≤3,000	28 (21,35)	
3,001–6,000	29 (23,37)	
6,001–10,000	31 (23,38)	
10,001–20,000	33 (25,39)	
≥20,001	33 (22,40)	
Wife's age at marriage (years)		<0.001
≤23	27 (21,35)	
24–35	31 (23,38)	
36–47	28 (16,35)	

P-value obtained from Chi-square and Kruskal-Wallis test

### Predictors of fertility decision model based on Theory of Planned Behavior (TPB) by structure equation model

#### Model fit indices

**Fig 2:**
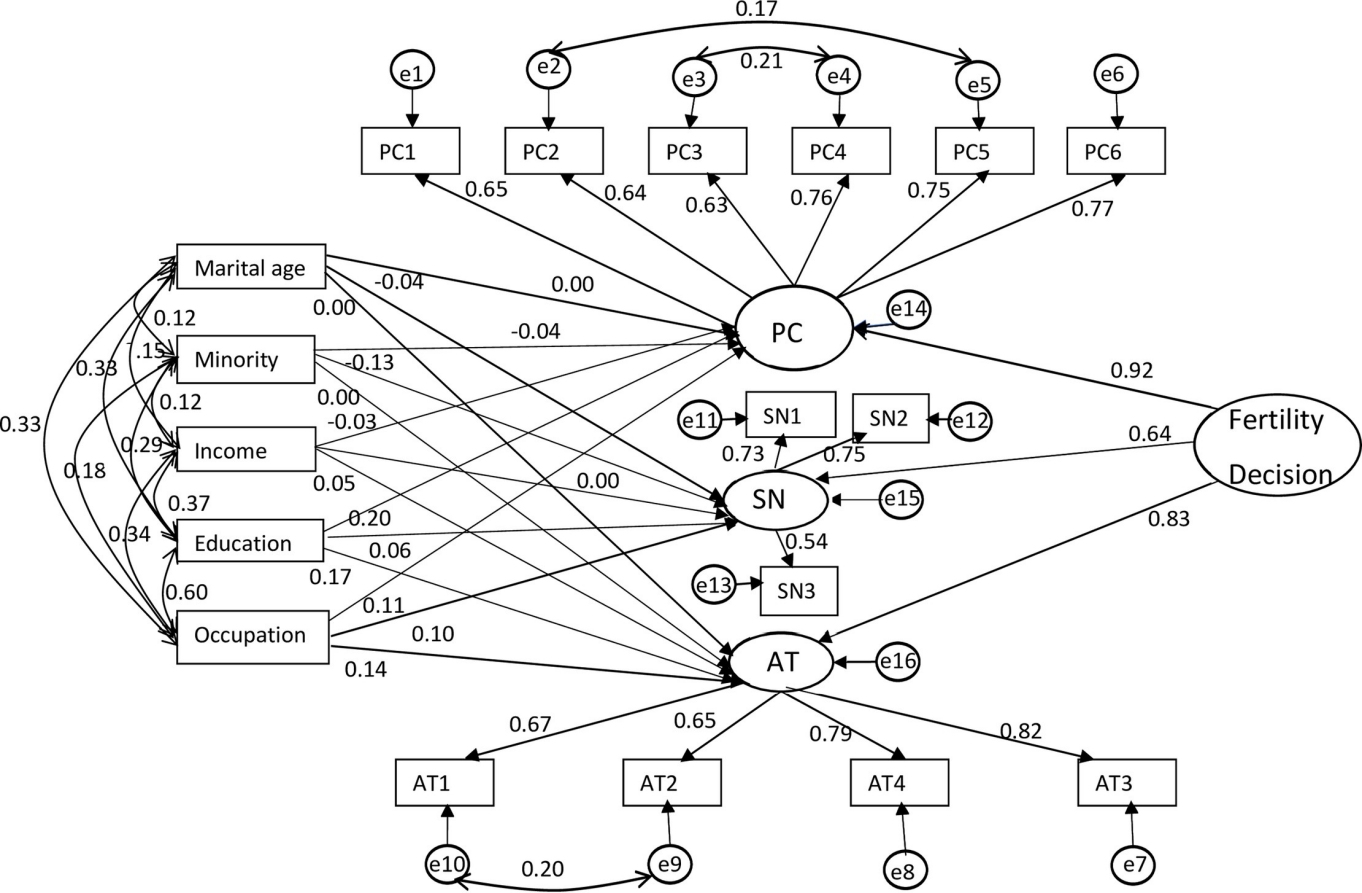
Final model of fertility intention based on Theory of Planned Behavior. PC: Perceived Behavior Control; PC1: The couple is ready to sacrifice the time and freedom for the baby; PC2: A suitable babysitter is available when the couple works outside; PC3: A lot of money will be spent to raise a (another) child; PC4: The couple has sufficient materials for child rearing; PC5: The couple’s family members can help them take care of the baby; PC6: The couple has a stable income; SN: Social Norms; SN1: The family should have a boy (or girl); SN2: His/her partner prefers to have a boy (or girl); SN3: The relatives and friends around the couple have already had two children; AT: Attitudes; AT1: The couple enjoys having a big family; AT2: The couple enjoys the fun of raising a child; AT3: Only healthy parents can have a child; AT4: Having a child can maintain a good relationship between the couple; e in a circle denotes measurement error of that latent variable.

The overall fit indices for the final structural equation model were as follows: the relative Chi-square = 3.868 (χ^2^/df), RMSEA = 0.045 (90% CI: 0.041, 0.050), AGFI = 0.949, and CFI = 0.966. All indices indicated that the final model fitted the data well. Fig 2 shows the structural model of the association between fertility decision with demographic characteristics.

#### Standardized regression weights

Table 4 shows standardized regression weights of all variables in the fertility decision model, based on the theory of planned behavior, obtained from the SEM. The strongest determining domain was perceived behavior control (β = 0.92, 95% CI: 0.89, 0.98), followed by attitudes (β = 0.83, 95% CI: 0.78, 0.87) and subjective norm (β = 0.64, 95% CI: 0.59, 0.69). Within each domain, perceived importance of having a stable income (PC6) (β = 0.77, 95% CI: 0.74, 0.79), subjective norm on the partner’s sex preference of the newborn child (SN2) (β = 0.75, 95%CI: 0.72, 0.80) and the attitude that only healthy parents can have a child (AT3) (β = 0.82, 95% CI: 0.78, 0.85) were the most important manifest variables.

**Table 4:** Standardized regression weights of parameters.

Endogenous variables	Exogenous variables	Estimate (β)	Lower	Upper
Fertility decision	Perceived behavior control	0.92	0.89	0.98
	Attitudes	0.83	0.78	0.87
	Subjective norm	0.64	0.59	0.69
Perceived behavior control	PC1	0.65	0.61	0.69
	PC2	0.64	0.60	0.68
	PC3	0.63	0.58	0.67
	PC4	0.76	0.72	0.79
	PC5	0.75	0.70	0.78
	PC6	0.77	0.73	0.79
Attitudes	Attitude1	0.66	0.62	0.71
	Attitude2	0.65	0.60	0.69
	Attitude3	0.82	0.78	0.85
	Attitude4	0.79	0.75	0.82
Subjective norm	SN1	0.73	0.68	0.78
	SN2	0.75	0.72	0.80
	SN3	0.54	0.46	0.59
Ethnic group	Subjective norm	-0.13	-0.18	-0.06
	Perceived behavior control	-0.04	-0.11	0.011
	Attitudes	0.00	-0.07	0.05
Education	Subjective norm	0.06	-0.02	0.17
	Perceived behavior control	0.20	0.14	0.27
	Attitudes	0.16	0.09	0.24
Wife's age at marriage	Subjective norm	-0.04	-0.11	0.03
	Perceived behavior control	-0.003	-0.06	0.06
	Attitudes	-0.004	-0.05	0.07
Employment status	Subjective norm	0.10	0.03	0.19
	Perceived behavior control	0.11	0.03	0.18
	Attitudes	0.13	0.07	0.20
Monthly income (RMB)	Subjective norm	0.004	-0.06	0.07
	Perceived behavior control	-0.03	-0.08	0.04
	Attitudes	0.05	-0.01	0.10

For explanation of the variable names, see Table 2

Ethnic group was significantly associated with the subjective norms towards fertility decision, with ethnic minority couples having less weight than Han couples (β = -0.13, 95% CI: -0.18, -0.06). Education was significantly associated with perceived behavior control and attitudes, but not with subjective norms. Couples with higher education had more perceived behavior control (β = 0.20, 95% CI: 0.14, 0.27) and attitudes (β = 0.83, 95% CI: 0.78, 0.87) than those with lower education. Employment status also had significant and positive relationship with all three domains of the fertility decision model.

## Discussion

This study followed the Theory of Planned Behavior (TPB) with the aim of advancing the understanding of determinants of fertility behavior among couples in Inner Mongolia, China. The strongest determinants of the fertility decision among Chinese couples were found to be in the domain of attitudes, which were that only healthy parents can have a child (AT3) (β = 0.82, 95% CI: 0.78, 0.85) and that a child can maintain a good relationship between the couples (AT4) (β = 0.79, 95% CI = 0.75, 0.82) reflecting that having a child cannot only affect the relationship between a couple, but also link with the health status of the couple. Infertility in China is very low. If young couples do not have children, there will be pressure from family members and friends. One study [35] showed that both positive and negative partner relationships had a negative effect on the timing of first as well as second and third births. Another study [36] suggested that childlessness might result in a decrease in quality of life and increase in marital discord and sexual dysfunction. Childlessness could also impose physical, psychological, emotional, and financial burdens. Our findings thus emphasize the role of children in Chinese families and call for public support for older couples who are childless.

Among the perceived behavior control variables, the perceived importance of having a stable income (β = 0.77, 95% CI: 0.73, 0.79) and having family members to help take care of the child (β = 0.75, 95% CI: 0.71, 0.78) had the highest effects on fertility decision. This indicates the high concerns on the costs for raising a child and awareness of the burden of childcare by couples. The cost of raising a child does not only depend on the financial costs, but also on the value of child in their parent’s view, which changes with time. Children became increasingly valued for their emotional worth rather than for their economic contribution in modern society of China, with compulsory education and schooling replacing work as the child’s primary social obligation [37]. Because of the inadequate infant-care social welfare system, it is common that the grandparents provide childcare for the third generations in China. Such situation makes the couples at childbearing age hesitate to have a (another) child [38]. Similar findings were found even in the context of a country with strong institutional support for childrearing such as Norway and Japan [26, 27, 39].

The perceived importance of having a suitable babysitter (PC2) shows the change in child-rearing practices of a family where young parents need others to help take care of their baby. In traditional Chinese culture, caring for the elders is the responsibility and obligation of the whole family, especially the children. However, in the Chinese modern society, the family structure relationship has shifted from the older-centered intergenerational relationships to the next generation as the center [40]. Older parents provide childcare for the three generations fostering a better relationship between generations [10]. This can explain why the couples in our study raised the issues of having a babysitter and family member to take care of their baby as the important determinants for their fertility behavior.

The strong effect of perceived importance of self-sacrifice of time and freedom for their baby (PC1) shows that with the development of society and an increase in people’s education level and self-consciousness, young couples no longer passively accept the birth of a child. Rearing a newborn baby needs a lot of time and energy by the parents; this not only increases the economic pressure on the entire family, but more importantly, devoted love of children needs enormous efforts from parents. Another reality is that the time allowed during statutory maternity leave has been found to be insufficient for taking care of a newborn child in China [41] and public kindergartens do not accept children less than three years of age [42]. Therefore, to have a child may have a great impact on a couple’s career advancement, so a couple’s career type and aspirations may have a major influence on their fertility intentions.

The sex preference of the newborn child was measured by two subjective norms (SN1: the preference of the respondent as representing the couple and SN2: the preference of his/her partner), which had significant effects on fertility decision. In contemporary China, gender preference is a sensitive issue, which is often described in the public and popular discourse as ‘‘traditional’, ‘backward’ and ‘feudal’ [10, 43]. In fact, preference for a son, a desire common among couples in India, Nepal, and Bangladesh [43, 44], is also common in China. China’s one-child policy, implemented in 1979 and recently abolished in January 2016, contributed to the country’s high sex ratio, the highest in the world. A higher proportion of males in their reproductive age was estimated at around 30 million in 2010 [45]. Preference for a son has changed in the modern Chinese society. Raising a son entails huge economic pressures, and people now realize that daughters, more than sons, can provide better emotional support for their parents. The advantages of having a daughter are increasingly being recognized by more couples, and more young couples have shifted their attention from reproducing a particular sex to the quality of child care [10]. In this study we did not specify the preference for a boy or girl in the questionnaire, but simply a sex preference, however our findings indicate that sex preference (SN1 and SN2) still matters in the decision by a couple to have a child and, in fact, had a greater impact than their relatives and friends already having two children (SN3), which, since the adoption of the universal two-child policy, has become more popular.

Apart from the above-mentioned determinants, ethnic minority couples had less weight than Han couples (β = -0.13, 95% CI: -0.18, -0.06). The fertility culture of Han Chinese prefer to have at least a son and multi children [46]. Compared with the fertility culture of Han, the minority Mongolians do not have such preferences [47].

Couples who were both employed perceived more behavior control compared with couples who had at least one self-employed. People’s increasing educational attainment and labor force participation has contributed to fertility decline in most developing countries [48]. The family role conflict theory asserts that there is a contradiction between work and family life, which are time conflicts, stress conflicts and behavior conflicts [49]. In the modern society of China, young couples put high values on educational attainment and career promotion, so they may worry that having a (another) child would be an obstacle and be an opportunity cost.

Although this study showed reasonable similarities with previous studies both internationally and in China, some limitations should be acknowledged. Our study aimed to describe the fertility situation in Inner Mongolia, therefore the generalizability of the findings to the whole China is limited. Although cross-sectional designs cannot reveal causal relationships, they can provide some points for further studies. Also, the discrepancy of fertility intentions between the first and second child was not specified.

## Conclusions

This study supports the assumption that use of the theory of planned behavior can explain the fertility decision-making model, which includes perceived behavior control, subjective norms, and attitudes. Due to the culture, minority couples are less affected by subjective norms. With improved education and employment, these couples may be more affected by perceived behavior control, subjective norms and positive attitudes towards to having a child in the future. Result of this study can help the Chinese government improve the fertility intention of its residents and the fertility rate of the country. We have the following recommendations. First, the government should provide equal access to basic public service as the expansion of basic education and infant care. Second, the government should provide adequate provisions for maternity protection and parental leave as an essential policy. Third, more consideration should be given to older ages couples who are involuntarily childless. Finally, the government should implement a supporting policy to reduce the time pressure and opportunity cost for couples who are conflicted by working and child-rearing. Sufficient parental leave for couples should be considered.

## Supporting information

S1 Dataset (CSV)
